# The promise of artificial intelligence-assisted radiotherapy for prostate cancer in Morocco: a transformational opportunity

**DOI:** 10.3389/fmed.2025.1577034

**Published:** 2025-09-08

**Authors:** Fadila Kouhen, Meryem Naciri, Hanae El Gouache, Nadia Errafiy, Abdelhak Maghous

**Affiliations:** ^1^Mohammed VI Faculty of Medicine, Mohammed VI University of Sciences and Health (UM6SS), Rabat, Morocco; ^2^Radiotherapy Department, International University Hospital Sheikh Khalifa, Casablanca, Morocco; ^3^Laboratory of Neurooncology, Oncogenetics, and Personalized Medicine, Casablanca, Morocco; ^4^Faculty of Medicine and Pharmacy Casablanca (FMPC), Hassan II University of Casablanca, Casablanca, Morocco; ^5^Mohammed V Military Teaching Hospital, Rabat, Morocco

**Keywords:** artificial intelligence, radiotherapy, prostate cancer, workflow optimization, Morocco

## Introduction

Prostate cancer is rapidly emerging as a significant public health concern in Morocco, with an incidence rate of 15 new cases per 100,000 men annually (1). This escalating burden is placing considerable strain on the healthcare system, which is equipped with only approximately 80 linear accelerators to serve a population of 37 million.

As one of the most frequently diagnosed cancers among Moroccan men, its management demands a highly precise approach, especially in radiotherapy, which remains a cornerstone for treating localized disease (2). However, despite its proven effectiveness, traditional radiotherapy faces significant challenges such as inconsistent tumor delineation, variability in treatment planning, and the risk of radiation-induced toxicity to surrounding healthy tissues. These obstacles are even more pronounced in Morocco, where access to specialized radiotherapy services is still limited, particularly in rural areas where 40% of the population resides.

AI is poised to transform prostate cancer treatment by improving radiotherapy precision (3). AI algorithms enhance tumor segmentation, treatment planning, and response prediction, enabling more personalized care (4, 5). While deep learning models and ANNs show superior accuracy globally, concerns remain about their applicability to Moroccan and African populations, as many models are trained on Western datasets (6).

The absence of locally validated AI solutions and standardized national radiotherapy guidelines for prostate cancer highlights the urgent need for context-specific research and tailored implementation strategies (7). Morocco's “Plan Cancer 2020–2029” prioritizes technological innovation, creating a unique opportunity for AI integration.

This article highlights the importance of integrating AI into prostate cancer radiotherapy in Morocco. It discusses AI's scientific principles, clinical applications, and challenges in a resource-limited healthcare system. Embracing AI can improve treatment accuracy, bridge gaps in cancer care, and enhance patient outcomes, making a strong case for its urgent implementation in the fight against prostate cancer.

### AI-powered automation in tumor segmentation and planning

Tumor segmentation, the delineation of tumors and surrounding healthy tissues on medical images (e.g., CT, MRI), is a critical yet time-consuming and error-prone step in radiotherapy (8). Accuracy in segmentation directly impacts treatment quality and patient outcomes. This challenge is particularly significant in Morocco, where a shortage of radiation oncology specialists further exacerbates the burden on the healthcare system.

AI, particularly deep learning models like CNNs, enhances segmentation accuracy. Arjmandi et al. showed the effectiveness of combining CNNs with Vision Transformers (ViT) in a study of 104 prostate cancer patients. Their CNN-based Segmentation Transformer model achieved high Dice Similarity Coefficients (DSCs), 91.75% for the prostate and over 95% for the bladder and femoral heads, outperforming traditional models (9).

Similarly, a large-scale Swedish study by Polymeri et al. (10) validated AI-assisted segmentation in prostate cancer radiotherapy planning (RTP) using 1,530 patient datasets (Table 1). The AI-generated contours showed strong concordance with manual delineations, achieving DSCs of 0.82 for the prostate, 0.95 for the bladder, and 0.88 for the rectum. Additionally, a real-world validation study by Palazzo et al. (11) demonstrated that AI-assisted contouring significantly reduced inter-observer variability and oncologist workload, reducing contouring time from 17 to 24 min manually to just 3–7 min with AI-assisted editing (*p* < 0.01).

**Table 1:** Overview of artificial intelligence applications in prostate radiotherapy treatment.

**Task**	**AI technique**	**First author (year)**	**Dataset size**	**Public/multi-institutional**	**Performance metrics**
Tumor segmentation	Hybrid CNN-ViT (VGG16-UNet-ViT with attention-based fusion)	Najmeh Arjmandi (2024)	104 patients	Multi-institutional (retrospective)	Prostate: 91.75%, bladder: 95.32%, rectum: 87.00%, RFH: 96.30%, LFH: 96.34%
Tumor segmentation	AI-based auto-contouring (model not specified)	Eirini Polymeri (2023)	1,530 patients	Multi-institutional (2006–2018)	Dice: prostate 0.82, bladder 0.95, rectum 0.88; mean SD: 1.7/0.7/1.1 mm; Hausdorff: 9.2/6.7/13.5 mm, respectively
Tumor segmentation	Commercial deep learning-based auto-segmentation system (architecture not specified)	Gabriele Palazzo (2023)	20 patients	Single center	Dice similarity coefficients (DSC): manual vs. auto (0.65–0.94), auto + edit (0.76–0.94); clinical score median: 4/5; time: reduced from 17–24 to 3–7 min (*p* < 0.01); improved consistency
Automated radiotherapy treatment planning	Hierarchical intelligent automatic treatment planning (HieVTPN)	Chenyang Shen (2021)	Prostate IMRT: 10 training cases, 5 validation cases, 59 testing cases; SBRT: 5 testing cases	Single center	Hierarchical intelligent automatic treatment planning (HieVTPN): IMRT: plan score 8.62 ± 0.83 (vs. VTPN 8.45 ± 0.48); SBRT: plan score 139.07 (vs. human plans 132.21); better scalability and explainability than prior VTPN
Prostate IMRT automatic treatment planning	DRL-based VTP using Q-learning with DVH input, ε-greedy policy, and GPU acceleration; integrated with Eclipse TPS via API	Damon Sprouts (2022)	50 test cases + 2 eclipse TPS deployment cases	Single center	Mean ProKnow score improved from 6.18 ± 1.75 to 8.14 ± 1.27 (max = 9); eclipse cases improved from 8 to 8.4 and 8.7; VTP mimicked human planning via iterative constraint adjustment
Prostate SBRT automatic treatment planning	DRL-based VTP using DVH input to adjust dose, volume, and weights; integrated with Eclipse TPS via API	Yin Gao (2023)	36 clinical cases (20 IMRT, 16 VMAT)	Single center	AAMD/RSS case: score 142.1/150 (3rd place, median 134.6); clinical: VTP vs. human scores—IMRT: 110.6 ± 6.5 vs. 110.4 ± 7.0; VMAT: 126.2 ± 4.7 vs. 125.4 ± 4.4; physicist-reviewed
Prostate VMAT machine parameter optimization (MPO)	RL-based policy network for 3D beam VMAT planning; integrated with TPS for automatic refinement	William T Hrinivich (2024)	136 patients	Single center	Execution: 3.3 ± 0.5 s (RL) + 77.4 ± 5.8 s (TPS); RL + TPS plans: *D*_max_ 63.2 ± 0.6 Gy vs. 63.9 ± 1.5 Gy (*p* = 0.061), rectum mean dose 17.4 ± 7.4 vs. 21.0 ± 6.0 (*p* = 0.024)
Prostate LDR brachytherapy plan generation	ML-based case-matching + stochastic optimization	Alexandru Nicolae (2017)	100 LDR cases (training + testing)	Single center	Planning time: ML 0.84 ± 0.57 min vs. BT 17.88 ± 8.76 min (*p* = 0.020); V150% 4% lower (*p* = 0.002, not clinically significant); expert likert scores: equivalent
Prostate LDR brachytherapy plan evaluation	ML-based implant planning algorithm (PIPA)	Alexandru Nicolae (2020)	41 patients	Single center	No significant differences in prostate D90%, V100%, rectum V100, D1cc between ML and manual; planning time ML 2.38 ± 0.96 min vs. manual 43.13 ± 58.70 min (*p* < < 0.05)
Brachytherapy treatment planning (prostate and cervix)	AI-based flexible optimization method (BRIGHT)	Leah R M Dickhoff (2024)	Prostate (*n* =12), Cervix (*n* = 36)	Single center	Finds multiple near-optimal plans with similar dose-volume criteria but different dose distributions; supports hospital-specific aims; improves adherence to EMBRACE-II protocol; enables fast plan adaptation
MRI-to-sCT generation for MRI-only radiotherapy in prostate cancer	Conditional GAN (Pix2Pix)	Safaa Tahri (2023)	90 patients	Multi-institutional	MAE (HU), *D*_99%_ CTV, *V*_95%_ PTV, *D*_max_ bladder/rectum, 3D gamma (1%/1 mm)
MRI-only planning for proton therapy in prostate cancer	Commercial sCT generator (MRI planner v2.3, likely DL-based)	Kajsa M. L. Fridström (2024)	10 patients	Multi-institutional	MAE (HU), gamma pass rates (1%/1 mm), range difference, DVH parameters (CTV/PTV, OARs)
CBCT-to-sCT translation for ART in prostate cancer	Transformer-based DL (SwinUNETR vs. CNN-based U-net in CycleGAN)	Yuhei Koike (2024)	260 patients	Single institution	MAE (HU), gamma pass rates, DVH deviation (< 1%)
sCT generation from MRI and CBCT for pelvic radiotherapy	Multi-domain GAN (StarGAN vs. CycleGAN)	Paritt Wongtrakool (2025)	53 pelvic cancer cases	Single institution	MAE (HU), dose difference (< 2%), gamma pass rate (>90%), qualitative anatomical preservation
Adaptative radiotherapy	Hybrid data augmentation + transformer-based segmentation + CNN regression	Jing Wang (2024)		Single institution	DSC up to 0.9789, HD95 ~1.8 mm, 3D centroid error < 0.33 mm, tracking latency 90 ms/frame, error < 1 mm under high noise, success rate ≈ 100%
Adaptative radiotherapy	Deep learning model for autocontouring (Annotate ART-Plan) (V1.8.3, TheraPanacea)	Marcel Nachbar (2024)	232 T2w-MRI datasets from 47 patients (1.5T Elekta Unity MR-Linac)	Single-institution	DSC: bladder 0.97 (best), penile bulb 0.73 (worst); 95% HD: bladder 2.7 mm (best), rectum 6.9 mm (worst); sDSC: rectum 0.94 (best), anal canal 0.68 (worst); 80% clinically acceptable
Adaptative radiotherapy	In-house trained nnU-Net (auto-segmentation)	Maximilian Lukas Konrad (2024)	15 prostate cancer patients	Single-institution	Mean delineation time reduced from 9.8 to 5.3 min; more consistent timing; fewer re-adaptations needed
Survival prediction and decision support in prostate cancer	ANN models: MLP, MLP-N, and LSTM; comparison with Cox regression	Kyo Chul Koo (2020)	7,267 patients (1988–2017)	Multi-institutional	LSTM had highest predictive power (Harrell's C-index); outperformed Cox regression for 5- and 10-year survival
External validation of AI-based survival calculator	LSTM ANN model (SCaP survival calculator)	Bumjin Lim (2021)	4,415 patients	Multi-institutional: 3 institutions	5-year AUCs: 0.962 (CRPC), 0.944 (CSS), 0.884 (OS); 10-year AUCs: 0.959, 0.928, 0.854; Cal ibration accurate at 5 years, underestimated at 10
Predict response to hormonal therapy in prostate cancer	3D multi-branch CNN-transformer (CNNFormer)	Ibrahim Abdelhalim (2024)	39 patients	Single center	Accuracy: 97.5%, sensitivity: 100%, specificity: 95.83%

In Morocco, where the shortage of radiation oncology specialists places immense pressure on the healthcare system, AI integration could be particularly impactful. Automating tumor delineation would not only alleviate the burden on specialists but also ensure more consistent and accurate contouring, reducing treatment delays and optimizing patient outcomes. Recent studies emphasize the need for locally validated AI models to account for regional anatomical variations and imaging protocols.

### AI and reinforcement learning for prostate cancer treatment planning in EBRT and brachytherapy

Following tumor segmentation, treatment planning constitutes a critical step in the radiotherapy workflow. It involves determining the optimal radiation dose and beam configurations to achieve effective tumor control while minimizing exposure to surrounding healthy tissues. Traditionally, this process is complex, highly individualized, and dependent on manual adjustments by experienced dosimetrists and radiation oncologists. In resource-constrained settings like Morocco, such workflows can be time-intensive (often 4–6 h per case), inconsistent, and vulnerable to human error.

Artificial Intelligence (AI), particularly Reinforcement Learning (RL), is emerging as a transformative solution to streamline and standardize treatment planning in both external beam radiotherapy (EBRT) and brachytherapy (12, 13). RL models learn through trial-and-error interactions with their environment, refining their strategies based on feedback to maximize treatment efficacy while minimizing toxicity (14).

Sprouts et al. introduced a Deep Reinforcement Learning (DRL)-based Virtual Treatment Planner (VTP) designed to optimize intensity-modulated radiation therapy (IMRT) plans for prostate cancer (15). Using Q-learning and dose-volume histogram (DVH) inputs, the VTP autonomously adjusted dosimetric constraints to enhance plan quality (16). A 2024 study validated this framework by applying DRL to volumetric modulated arc therapy (VMAT), achieving comparable target coverage (63.2 ± 0.6 Gy) while reducing the mean rectal dose by 17% compared to clinical plans. When integrated with the Eclipse treatment planning system, the VTP improved average plan scores from 6.18 to 8.14 across 50 testing cases (17).

Recent developments have further improved training efficiency by 40% through the introduction of DVH-based embedding layers, enabling real-time adaptation to anatomical variability. In Moroccan settings where access to advanced planning technologies like IMRT may be limited, the integration of RL-based 3D-conformal planning tools could approximate high-quality dose distributions while reducing planning time to 1–2 h. This technology not only improves consistency and quality but also democratizes access to advanced planning capabilities across diverse treatment centers (18, 19).

In parallel, AI applications in prostate brachytherapy are also demonstrating significant clinical promise. Low-dose-rate (LDR) brachytherapy is a highly targeted approach for localized prostate cancer but involves intricate planning to determine seed placement and dose distribution. Traditionally reliant on expert intervention, brachytherapy planning can be both time-consuming and variable.

A Canadian study demonstrated that a machine learning (ML) algorithm could generate clinically equivalent LDR brachytherapy plans in just 0.84 min, compared to 17.88 min for expert-driven plans (20). These AI-generated plans achieved comparable target coverage, organ-at-risk (OAR) sparing, and implant confidence, with only a 4% lower prostate V150% a non-significant difference. Expert reviewers were unable to distinguish between AI-generated and human-created plans (20, 21).

Further advances include the BRIGHT AI system, which automatically generates multiple near-optimal plans, allowing clinicians to select the best trade-off between tumor coverage and healthy tissue preservation (22). The integration of deep reinforcement learning into brachytherapy workflows enables real-time constraint optimization and adaptive planning, supporting a synergistic relationship between human expertise and machine intelligence.

Despite the automation potential, human oversight remains essential to balance clinical nuances and anatomical variability, especially when navigating trade-offs between target dose escalation and rectal or urethral sparing. In resource-limited contexts, AI can significantly reduce clinician workload while ensuring high-quality, personalized treatment planning, even in the absence of highly specialized staff (23).

In Morocco, the integration of AI-driven planning tools across both EBRT and brachytherapy presents an unprecedented opportunity to enhance care equity, efficiency, and precision. RL-based systems can standardize workflows, reduce planning times by over 60%, and elevate the overall quality of radiotherapy services, especially in institutions lacking full-time medical physicists or IMRT infrastructure. AI thus offers not just automation, but augmentation of clinical expertise, ultimately improving access to safe and effective prostate cancer treatment nationwide (24, 25).

## Image-to-image translation and synthetic imaging in prostate radiotherapy

Artificial intelligence (AI), especially deep learning, has revolutionized medical image translation across modalities, significantly impacting radiation oncology (26). Accurate imaging is essential for precise treatment planning and delivery, particularly in prostate cancer radiotherapy. Traditionally, computed tomography (CT) has been the standard imaging modality for planning because it provides electron density information necessary for accurate dose calculation. However, magnetic resonance imaging (MRI) offers superior soft tissue contrast, enabling more precise tumor and organ-at-risk delineation (27, 28). This dual-modality approach, requiring both MRI and CT, introduces complexities including registration errors, increased patient time, and resource demands.

To overcome these challenges, AI-driven techniques have been developed to generate synthetic CT (sCT) images directly from MRI data (29). This innovation enables MR-only radiotherapy workflows by providing CT-equivalent images necessary for dose calculation without acquiring separate CT scans. Deep learning models such as U-Net architectures, Cycle-Consistent Generative Adversarial Networks (CycleGANs), and attention-guided GANs have been employed to achieve this (30–32). These networks are trained on datasets of paired or unpaired MRI and CT images to predict realistic Hounsfield Unit (HU) values on MRI scans, allowing accurate dose calculations.

A multi-center study by S tahri et al. demonstrated that a generic 2D conditional GAN (Pix2Pix) model produced sCT images from T2-weighted MRI with dosimetric accuracy comparable to models trained specifically for individual centers (33). The model yielded consistent mean absolute HU errors across pelvic structures and dose deviations under 1 Gy for the clinical target volume, with no significant differences in stringent gamma analysis metrics. This robustness suggests its practical potential for routine clinical use.

Further, retrospective studies assessing proton therapy dose calculations based on MRI-derived sCT showed minimal differences compared to planning CT-based doses for both photon and proton plans (34).

Although gamma pass rates were slightly lower for proton therapy, they remained within clinically acceptable thresholds, and proton range deviations averaged only 1.0 mm, indicating negligible clinical impact.

Clinically, this MRI-to-sCT approach has been integrated into MR-Linac systems such as Elekta Unity and ViewRay MRIdian, enabling real-time adaptive radiotherapy with MR-only planning. This is particularly advantageous in prostate cancer, where precise delineation of organs-at-risk like the bladder and rectum allows for reduction of planning target volume margins from 7–10 to 3–5 mm, potentially reducing toxicity (35).

In low- and middle-income countries (LMICs) such as Morocco, where access to MR-Linac technology is limited, leveraging standard MRI simulators combined with AI-based sCT generation offers a cost-effective pathway to MRI-guided radiotherapy. This reduces reliance on dual imaging, streamlines workflows, and enhances treatment precision and efficiency.

Similarly, cone-beam computed tomography (CBCT) is widely used in image-guided radiotherapy (IGRT) for patient positioning, but its inherent image quality limitations such as scatter noise, beam hardening artifacts, and inaccurate HU values have historically restricted its use for dose recalculation and adaptive planning. AI models have been developed to convert CBCT images into synthetic CT scans of planning quality, overcoming these limitations and enabling adaptive radiotherapy on standard linear accelerators (36, 37).

Approaches including CycleGANs trained on unpaired CBCT-CT datasets, 3D residual convolutional neural networks (CNNs) capturing volumetric context, and dual-input models combining CBCT with planning CT or anatomical contours have proven effective (38, 39). These techniques reduce artifacts, restore soft tissue contrast, and calibrate HU values, producing images suitable for accurate dose calculation.

In prostate cancer, AI-generated sCTs from daily CBCT enable clinicians to adapt treatment to anatomical changes such as bladder and rectal filling or prostate motion exceeding 5 mm. This adaptation improves target coverage and treatment precision.

A retrospective study with 260 patients found that a transformer-based SwinUNETR model outperformed conventional U-net architectures within a CycleGAN framework, achieving lower mean absolute HU errors and dose deviations under 1% (40). Another study comparing StarGAN and CycleGAN models showed StarGAN better preserved anatomical structures qualitatively, while both achieved clinically acceptable dosimetric accuracy with dose differences within 2% and gamma passing rates above 90% (37).

For resource-constrained settings like Morocco, where MR-guided adaptive radiotherapy remains limited, AI-powered CBCT-to-sCT conversion offers a practical, scalable solution to implement daily adaptive radiotherapy using existing linear accelerators. This innovation promises to improve treatment accuracy, optimize resource use, and ultimately enhance prostate cancer outcomes.

### Real-time tumor tracking and adaptive radiotherapy

In Morocco, radiotherapy resources are often constrained by high patient volumes, leading to potential delays and a reduction in treatment quality.

Prostate motion, induced by physiological factors such as bladder filling or rectal gas, further complicates this issue, as even minor displacements during treatment can compromise the precision of radiation delivery. This can affect tumor control and compromise the safety of surrounding tissues. AI technologies offer a promising solution to address this issue by enabling real-time tumor tracking with sub-millimeter accuracy, allowing adaptive radiotherapy where treatment plans are adjusted dynamically based on tumor position (41, 42).

In recent studies, advancements have been made in the development of AI-driven tools for adaptive radiotherapy. Nachbar et al. created an AI-based auto contouring model for online adaptive MR-guided radiotherapy using the 1.5 T MR-Linac system (43). This model achieved clinically acceptable contours in 80% of cases and required only minor adjustments in 16% of cases. It demonstrated high accuracy in segmenting structures like the bladder and rectum, with quantitative evaluations indicating excellent performance, making it suitable for future clinical implementation in MR-guided adaptive radiotherapy workflows.

Further research has evaluated the feasibility and time gains of AI-based delineation tools in daily prostate cancer radiotherapy. A study involving 15 consecutive prostate cancer patients treated with a 1.5 T MRI-Linac found that AI-based delineation reduced contouring time from 9.8 to 5.3 min, with lower variance in delineation time throughout the treatment course (44). The AI-based workflow also resulted in fewer instances of readaptation due to tumor motion, demonstrating the efficiency and time-saving potential of AI tools in enhancing radiotherapy processes.

This integration would be particularly beneficial in advanced treatment modalities like stereotactic body radiotherapy (SBRT), where precision is paramount. AI's ability to track even subtle tumor movements ensures that high doses of radiation are delivered precisely to the tumor, minimizing exposure to nearby organs at risk.

In Morocco, with its advanced healthcare and growing tech investment, AI models could reduce treatment complications, especially in busy settings. Despite economic progress, regional disparities and high patient loads remain challenges. Additionally, Moroccan patients value family support and personalized care. AI could assist clinicians by offering data-driven insights, enabling tailored treatment plans that align with patients' needs and preferences.

### AI in predictive analytics for disease progression and treatment response

AI is revolutionizing oncology by predicting tumor response and survival outcomes, enhancing clinical decision-making and personalized treatment. Recent advancements, like the Clinical Histopathology Imaging Evaluation Foundation (CHIEF) model, show AI's ability to predict survival, molecular profiles, and treatment response with 94% accuracy, outperforming traditional methods by up to 36%. AI-driven models, including ANNs and deep learning, offer superior accuracy, enabling more precise prognostic assessments in cancer care (45).

Koo et al. (46) developed an online support tool using a long short-term memory (LSTM) artificial neural network (ANN) model to predict survival outcomes for prostate cancer (PCa) patients. The model was trained using data from 7,267 cases and 19 clinicopathological covariates, significantly outperforming traditional Cox-proportional hazards regression models. The LSTM model demonstrated enhanced predictive power for 5- and 10-year progression to castration-resistant prostate cancer (CRPC)-free survival, cancer-specific survival (CSS), and overall survival (OS). These findings highlight AI's ability to refine individualized treatment planning by providing more accurate prognostic estimates than conventional methodologies.

Similarly, the SCaP Survival Calculator, another AI-powered tool utilizing an LSTM ANN model, was externally validated in a cohort of 4,415 PCa patients diagnosed between April 2005 and November 2018 across three institutions (47). The model effectively predicted survival outcomes, including CRPC-free survival, CSS, and OS, with area under the curve (AUC) values of 0.962, 0.944, and 0.884 for 5-year outcomes, and 0.959, 0.928, and 0.854 for 10-year outcomes, respectively. The superior discrimination ability of the SCaP model underscores AI's potential in enhancing clinical risk stratification and treatment decision-making.

AI's role in predicting tumor response extends beyond survival modeling. By integrating multi-omics data, imaging biomarkers, and real-world clinical variables, AI-driven models can enhance precision oncology, allowing for better patient stratification and treatment personalization. Future advancements may incorporate genomic and radiomic features to further refine predictive accuracy, ultimately transforming prostate cancer management and improving patient outcomes.

In Morocco, the integration of AI-driven predictive tools presents both a challenge and an opportunity. The country faces a lack of comprehensive local clinical guidelines, particularly for precision oncology and advanced treatment strategies. As a result, oncologists often rely on international recommendations that may not fully align with the genetic, epidemiological, and healthcare infrastructure specific to Morocco.

The adoption of AI models like the SCaP Survival Calculator could bridge this gap by providing data-driven, personalized insights tailored to local patient populations.

## Conclusion

AI holds immense potential to transform prostate cancer care in Morocco. By enhancing tumor segmentation, optimizing treatment planning, enabling real-time tumor tracking, and predicting side effects and disease progression, AI can significantly improve the accuracy, efficiency, and personalization of radiotherapy. However, to fully realize these benefits, Morocco must invest in infrastructure, financial support, and workforce training. With strategic planning and international collaboration, AI could revolutionize prostate cancer treatment in Morocco, improving patient outcomes and setting a global example for AI integration in oncology.
